# Supplementary material for: National-scale acoustic monitoring of avian biodiversity and migration
Source: Commun Biol. 2026 May 30;9:887. doi: 10.1038/s42003-026-10389-4 (PMC13328752; doi:10.1038/s42003-026-10389-4)
Supplement: Supplementary file 1 — Supplementary Information [file 42003_2026_10389_MOESM1_ESM.pdf]

Supplementary Information

Supplementary Figure 1. Acoustic recorder uptime percent for each site across the study period. Grey areas indicate no data availability.

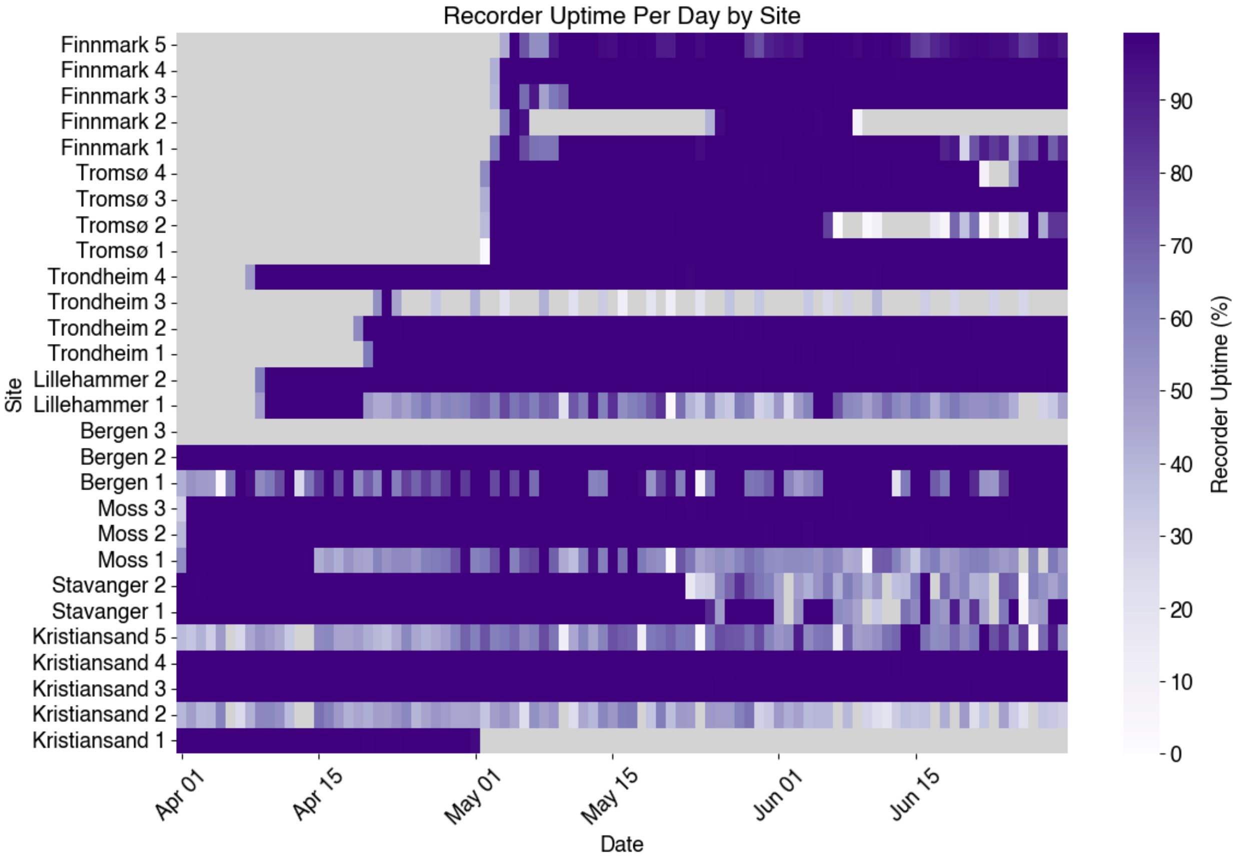

Supplementary Table 1. Total detections of Willow Warbler (WW), Spotted Flycatcher (SF), and Common Chiffchaff (CC) by study region and monitoring method.

Region	Species	BBS Detections	eBird Detections	Audio Detections
Kristiansand	WW	95	34	425
Bergen	WW	65	1	2431
Trondheim	WW	88	5	158
Tromsø	WW	36	1	124
Stavanger	WW	48	2	29
Moss	WW	88	18	41
Lillehammer	WW	58	0	3
Kristiansand	SF	4	4	97
Bergen	SF	2	2	88
Trondheim	SF	3	0	4

Tromsø	SF	0	0	0
Stavanger	SF	0	0	8
Moss	SF	13	10	1
Lillehammer	SF	0	0	0
Kristiansand	CC	9	2	1
Bergen	CC	8	2	339
Trondheim	CC	51	9	151
Tromsø	CC	0	5	0
Stavanger	CC	2	2	0
Moss	CC	6	7	0
Lillehammer	CC	3	0	0

Supplementary Table 2. Regional total bird detections for three monitoring methods (PAM, BBS, and eBird) for April through June 2022. Detections are shown before and after data filtering steps. Species richness information is shown for filtered detections. For PAM, the "region detections unfiltered" column represents the total number audio detections per region for 57 species for which BirdNET was shown to have at least 80% precision; the "region detections filtered" column represents detections filtered for high model confidence (≥0.80). For BBS, the "region detections unfiltered" column represents all detections for WW, SF, and CC only within each region, as only data for these species were provided by BBS. For BBS, the "region detections filtered" column was additionally filtered to detections within the altitude range of our recorders and for detections in forested areas. For eBird, the "region detections unfiltered" column shows total species detections for all species after dropping any duplicates stemming from group surveys. For eBird, the "region detections filtered" column was further filtered to remove high effort surveys, surveys not within forested regions, non-complete surveys, and surveys not in the altitude range of recorders. See the Methods section for more details on filtering. The "filtered species richness" and "filtered species list" columns account for species present in the filtered data.

Survey	Region	Region Detections Unfiltered	Region Detections Filtered	Filtered Species Richness	Filtered Species List
PAM	Tromsø	9133	1694	21	['Brambling', 'Fieldfare', 'Long-eared Owl', 'Redwing', 'Willow Warbler', 'Dunnock', 'Common Redpoll', 'Reed Bunting', 'Eurasian Siskin', 'Common Sandpiper', 'Willow Tit', 'Eurasian Woodcock', 'Common Raven', 'Great Tit', 'Eurasian Bullfinch', 'European Pied Flycatcher', 'Yellowhammer', 'European Greenfinch', 'Marsh Tit', 'White Wagtail', 'Hooded Crow']
PAM	Kristiansand	30349	5172	49	['Common Sandpiper', 'Common Swift', 'Dunnock', 'Eurasian Siskin', 'Goldcrest', 'Willow Warbler', 'Coal Tit', 'Eurasian Bullfinch', 'Willow Tit', 'Eurasian Treecreeper', 'Great Spotted Woodpecker', 'European Robin', 'European Greenfinch', 'Eurasian Nuthatch', 'Barn Swallow', 'Hazel Grouse', 'Eurasian Blue Tit', 'Eurasian Jay', 'Crested Tit', 'Eurasian Woodcock', 'Spotted Flycatcher', 'European Pied Flycatcher', 'Great Tit', 'Greater Whitethroat', 'Common Chaffinch', 'Common Redpoll', 'Eurasian Magpie', 'Common Raven', 'Redwing', 'Common Crane', 'Common Cuckoo', 'Eurasian Blackbird', 'Song Thrush', 'Fieldfare', 'Eurasian Green Woodpecker', 'Green Sandpiper', 'Marsh Tit', 'Eurasian Oystercatcher', 'Eurasian Blackcap', 'Garden Warbler', 'Yellowhammer', 'European Goldfinch', 'White Wagtail', 'Long-eared Owl', 'Brambling', 'Common Wood-Pigeon', 'Eurasian Wren', 'Black Woodpecker', 'Common Chiffchaff']
PAM	Trondheim	9035	1329	40	['European Robin', 'Common Chiffchaff', 'Great Tit', 'Eurasian Bullfinch', 'Eurasian Blackbird', 'Song Thrush', 'Hazel Grouse', 'Eurasian Siskin', 'Brambling', 'Common Chaffinch', 'Eurasian Blue Tit', 'Marsh Tit', 'Great Spotted Woodpecker', 'Barn Swallow', 'Eurasian Wren', 'Dunnock', 'Fieldfare', 'Redwing', 'Eurasian Woodcock', 'Long-eared Owl', 'Willow Tit', 'Common Raven', 'Willow Warbler', 'Eurasian Nuthatch', 'Goldcrest', 'Spotted Flycatcher', 'Eurasian Green Woodpecker', 'Eurasian Treecreeper', 'Common Sandpiper', 'Coal Tit', 'Mew Gull', 'Hooded Crow', 'Eurasian Blackcap', 'European Pied

					Flycatcher', 'Black Woodpecker', 'Green Sandpiper', 'European Goldfinch', 'Common Crane', 'European Greenfinch', 'Eurasian Jay']
PAM	Bergen	68241	12497	45	['Eurasian Woodcock', 'Eurasian Blackbird', 'Eurasian Wren', 'Eurasian Siskin', 'Marsh Tit', 'Eurasian Blue Tit', 'European Greenfinch', 'Eurasian Treecreeper', 'Common Chiffchaff', 'Barn Swallow', 'Willow Warbler', 'Willow Tit', 'Eurasian Nuthatch', 'Dunnock', 'European Robin', 'Spotted Flycatcher', 'Mew Gull', 'Eurasian Bullfinch', 'Gray Heron', 'Eurasian Blackcap', 'Song Thrush', 'Crested Tit', 'European Pied Flycatcher', 'Coal Tit', 'Eurasian Oystercatcher', 'Great Spotted Woodpecker', 'Eurasian Jay', 'European Goldfinch', 'Great Tit', 'Redwing', 'Common Chaffinch', 'Goldcrest', 'Hazel Grouse', 'Common Raven', 'Hooded Crow', 'Eurasian Magpie', 'Fieldfare', 'Common Redpoll', 'Eurasian Green Woodpecker', 'Common Sandpiper', 'Common Wood-Pigeon', 'Green Sandpiper', 'Brambling', 'Long-eared Owl', 'White Wagtail']
PAM	Stavanger	9894	1459	37	['Redwing', 'Common Cuckoo', 'Common Sandpiper', 'Eurasian Woodcock', 'Black Woodpecker', 'Eurasian Green Woodpecker', 'Common Raven', 'Eurasian Nuthatch', 'Willow Warbler', 'Eurasian Siskin', 'Eurasian Blackbird', 'Spotted Flycatcher', 'Common Chaffinch', 'European Robin', 'Marsh Tit', 'Willow Tit', 'Common Crane', 'Great Tit', 'Fieldfare', 'Eurasian Bullfinch', 'Eurasian Blackcap', 'Long-eared Owl', 'Eurasian Blue Tit', 'Eurasian Treecreeper', 'Eurasian Jackdaw', 'Garden Warbler', 'Eurasian Magpie', 'White Wagtail', 'Graylag Goose', 'European Pied Flycatcher', 'Eurasian Wren', 'Eurasian Jay', 'Dunnock', 'Song Thrush', 'Common Redpoll', 'Yellowhammer', 'Brambling']
PAM	Moss	7171	791	39	['Common Wood-Pigeon', 'Eurasian Blackcap', 'Great Spotted Woodpecker', 'Eurasian Blackbird', 'Eurasian Blue Tit', 'Eurasian Siskin', 'Black Woodpecker', 'European Robin', 'Crested Tit', 'Great Tit', 'Eurasian Woodcock', 'Spotted Flycatcher', 'Song Thrush', 'Eurasian Treecreeper', 'Eurasian Wren', 'Goldcrest', 'Eurasian Bullfinch', 'Marsh Tit', 'Willow Warbler', 'Common Crane', 'Dunnock', 'Common Cuckoo', 'Coal Tit', 'Common Chaffinch', 'Eurasian Magpie', 'Eurasian Oystercatcher', 'Eurasian Jay', 'Green Sandpiper', 'Willow Tit', 'Eurasian Green Woodpecker', 'White Wagtail', 'Eurasian Nuthatch', 'European Pied Flycatcher', 'Common Swift', 'Common Sandpiper', 'Reed Bunting', 'Common Raven', 'Redwing', 'Brambling']
PAM	Finnmark	1306	126	6	['Brambling', 'Redwing', 'Eurasian Bullfinch', 'Hooded Crow', 'Common Redpoll', 'Willow Tit']
PAM	Lillehammer	397	41	10	['Eurasian Siskin', 'Willow Tit', 'Eurasian Bullfinch', 'Redwing', 'European Robin', 'Eurasian Nuthatch', 'Goldcrest', 'Eurasian Woodcock', 'Willow Warbler', 'Dunnock']
BBS	Moss	151	108	N/A	N/A
BBS	Lillehammer	202	68	N/A	N/A
BBS	Trondheim	245	141	N/A	N/A
BBS	Kristiansand	113	107	N/A	N/A
BBS	Stavanger	81	43	N/A	N/A
BBS	Bergen	160	86	N/A	N/A
BBS	Tromsø	45	32	N/A	N/A
BBS	Finnmark	41	24	N/A	N/A
eBird	Moss	3036	135	14	['Eurasian Blue Tit', 'Common Wood-Pigeon', 'Brambling', 'Common Chaffinch', 'Eurasian Nuthatch', 'Barn Swallow', 'Eurasian Blackcap', 'Black Woodpecker', 'Arctic Loon', 'Barnacle Goose', 'Black Redstart', 'Common Swift', 'Eurasian Magpie', 'Eurasian Linnet']
eBird	Lillehammer	324	12	4	['Eurasian Blue Tit', 'Common Greenshank', 'Common Merganser', 'Common Chaffinch']
eBird	Kristiansand	867	150	8	['Black Woodpecker', 'Coal Tit', 'Eurasian Blackcap', 'Eurasian Blue Tit', 'Common Buzzard', 'Common Chaffinch', 'Bank Swallow', 'Barn Swallow']
eBird	Stavanger	1197	45	7	['Eurasian Blackcap', 'Eurasian Blue Tit', 'Common Chaffinch', 'Common Eider', 'Barn Swallow', 'Common Buzzard', 'Common Chiffchaff']
eBird	Bergen	318	12	3	['Eurasian Blue Tit', 'Barn Swallow', 'Common Chaffinch']
eBird	Trondheim	735	60	10	['Black Woodpecker', 'Eurasian Blue Tit', 'Brambling', 'Canada Goose', 'Coal Tit', 'Barn Swallow', 'Black-headed Gull', 'Eurasian Blackcap', 'Common Crane', 'Common Chaffinch']
eBird	Tromsø	228	15	3	['Eurasian Blue Tit', 'Brambling', 'Meadow Pipit']
eBird	Finnmark	534	81	12	['Arctic Warbler', 'Eurasian Blue Tit', 'Bohemian Waxwing', 'Brambling', 'Common Chaffinch', 'Common Goldeneye', 'Common Redstart', 'Fieldfare', 'Gray-headed Chickadee', 'Common Scoter', 'Bluethroat', 'Song Thrush']

Supplementary Table 3: Results of expert ornithologist validation of BirdNET detections. At least 50 random detections, with a model confidence of at least 0.80, were reviewed per species.

Species	BirdNET Correct Percent	BirdNET Incorrect Percent	BirdNET Unsure Percent
Barn Swallow	100	0	0
Barnacle Goose	80	16	4
Black Grouse	0	100	0
Black Woodpecker	88	10	2
Boreal Owl	0	98	2
Brambling	98	0	2
Coal Tit	98	0	2
Common Buzzard	6	60	34
Common Chaffinch	100	0	0
Common Chiffchaff	100	0	0
Common Crane	80	18	2
Common Cuckoo	100	0	0
Common Raven	95	2	3
Common Redpoll	100	0	0
Common Sandpiper	100	0	0
Common Swift	100	0	0
Common Wood-Pigeon	100	0	0
Crested Tit	98	0	2
Dunnock	98	0	2
Eurasian Blackbird	100	0	0
Eurasian Blackcap	100	0	0
Eurasian Blue Tit	100	0	0
Eurasian Bullfinch	81	15	4
Eurasian Curlew	47	47	6
Eurasian Golden Oriole	0	100	0
Eurasian Green Woodpecker	100	0	0
Eurasian Jackdaw	96	0	4
Eurasian Jay	96	2	2
Eurasian Kestrel	76	0	24
Eurasian Magpie	100	0	0
Eurasian Nuthatch	98	0	2
Eurasian Oystercatcher	100	0	0
Eurasian Pygmy-Owl	0	100	0
Eurasian Siskin	100	0	0
Eurasian Three-toed Woodpecker	2	94	4

Eurasian Tree Sparrow	76	0	24
Eurasian Treecreeper	87	0	13
Eurasian Woodcock	100	0	0
Eurasian Wren	99	1	0
European Goldfinch	100	0	0
European Greenfinch	99	0	1
European Pied Flycatcher	84	10	6
European Robin	99	0	1
Fieldfare	98	2	0
Garden Warbler	100	0	0
Goldcrest	100	0	0
Gray Heron	86	14	0
Graylag Goose	98	1	1
Great Bittern	0	100	0
Great Spotted Woodpecker	95	0	5
Great Tit	100	0	0
Greater Whitethroat	100	0	0
Green Sandpiper	100	0	0
Hawfinch	51	18	31
Hazel Grouse	100	0	0
Herring Gull	98	0	2
Hooded Crow	98	0	2
Long-eared Owl	98	2	0
Long-tailed Tit	62	0	38
Marsh Tit	92	0	8
Mew Gull	100	0	0
Mistle Thrush	0	0	100
Parrot Crossbill	0	0	100
Red Crossbill	0	0	100
Red-breasted Flycatcher	0	100	0
Red-throated Loon	0	100	0
Redwing	85	1	15
Reed Bunting	100	0	0
Rook	10	10	80
Song Thrush	100	0	0
Spotted Flycatcher	96	1	3
Tawny Owl	65	27	8
Tree Pipit	62	26	12
Western Capercaillie	0	100	0

White Wagtail	100	0	0
Whooper Swan	0	100	0
Willow Tit	100	0	0
Willow Warbler	100	0	0
Wood Lark	0	100	0
Yellowhammer	100	0	0

Supplementary Table 4. Audio Species Distribution Model (aSDM) model hyperparameters, as well as test and train set mean absolute error (MAE) for Willow Warbler and Spotted Flycatcher. Predicted aSDM values range from 0.0 to 1.0. One Random Forest model was trained for each species and hyperparameters were chosen over 1000 iterations of randomized parameters to optimize the test set MAEs.

Species	Train MAE	Test MAE	Random Forest Parameters
Willow Warbler (*Phylloscopus trochilus*)	0.0939	0.188	{'n_estimators': 185, 'min_samples_split': 5, 'min_samples_leaf': 1, 'max_features': None, 'max_depth': 50, 'bootstrap': True}
Spotted Flycatcher (*Muscicapa striata*)	0.0311	0.0739	{'n_estimators': 329, 'min_samples_split': 2, 'min_samples_leaf': 1, 'max_features': None, 'max_depth': None, 'bootstrap': True}
