# Supplementary material for: National-scale acoustic monitoring of avian biodiversity and migration
Source: Commun Biol. 2026 May 30;9:887. doi: 10.1038/s42003-026-10389-4 (PMC13328752; doi:10.1038/s42003-026-10389-4)
Supplement: Supplementary file 2 — Reporting Summary [file 42003_2026_10389_MOESM2_ESM.pdf]

nature portfolio

Corresponding author(s): I Avery Bick
Sarab S Sethi

Last updated by author(s): Feb 7, 2026

Reporting Summary

Nature Portfolio wishes to improve the reproducibility of the work that we publish. This form provides structure for consistency and transparency in reporting. For further information on Nature Portfolio policies, see our Editorial Policies and the Editorial Policy Checklist.

Statistics

For all statistical analyses, confirm that the following items are present in the figure legend, table legend, main text, or Methods section.

n/a	Confirmed	
☐	☒	The exact sample size (*n*) for each experimental group/condition, given as a discrete number and unit of measurement
☐	☒	A statement on whether measurements were taken from distinct samples or whether the same sample was measured repeatedly
☐	☒	The statistical test(s) used AND whether they are one- or two-sided<br>*Only common tests should be described solely by name; describe more complex techniques in the Methods section.*
☐	☒	A description of all covariates tested
☐	☒	A description of any assumptions or corrections, such as tests of normality and adjustment for multiple comparisons
☐	☒	A full description of the statistical parameters including central tendency (e.g. means) or other basic estimates (e.g. regression coefficient) AND variation (e.g. standard deviation) or associated estimates of uncertainty (e.g. confidence intervals)
☐	☒	For null hypothesis testing, the test statistic (e.g. *F*, *t*, *r*) with confidence intervals, effect sizes, degrees of freedom and *P* value noted<br>*Give P values as exact values whenever suitable.*
☒	☐	For Bayesian analysis, information on the choice of priors and Markov chain Monte Carlo settings
☒	☐	For hierarchical and complex designs, identification of the appropriate level for tests and full reporting of outcomes
☐	☒	Estimates of effect sizes (e.g. Cohen's *d*, Pearson's *r*), indicating how they were calculated

Our web collection on statistics for biologists contains articles on many of the points above.

Software and code

Policy information about availability of computer code

Data collection	Bioacoustic data collection was performed using the open source Bugg hardware and software, available at https://github.com/bugg-resources
Data analysis	All data analysis was performed in Python, particularly using pandas, geopandas, and sklearn. All data and code are available at doi.org/10.5281/zenodo.11238950

For manuscripts utilizing custom algorithms or software that are central to the research but not yet described in published literature, software must be made available to editors and reviewers. We strongly encourage code deposition in a community repository (e.g. GitHub). See the Nature Portfolio guidelines for submitting code & software for further information.

Data

Policy information about availability of data

All manuscripts must include a data availability statement. This statement should provide the following information, where applicable:

- Accession codes, unique identifiers, or web links for publicly available datasets
- A description of any restrictions on data availability
- For clinical datasets or third party data, please ensure that the statement adheres to our policy

All code, manual survey data, acoustic detections, and ecoclimatic covariates required to generate each figure in this study are available at doi.org/10.5281/

zenodo.11238950. Random noise of up to 0.01 degrees has been added to all coordinates of recording stations and Norwegian Breeding Bird survey points to maintain the confidentiality and data integrity of the survey.

Research involving human participants, their data, or biological material

Policy information about studies with human participants or human data. See also policy information about sex, gender (identity/presentation), and sexual orientation and race, ethnicity and racism.

Reporting on sex and gender	N/A
Reporting on race, ethnicity, or other socially relevant groupings	N/A
Population characteristics	N/A
Recruitment	N/A
Ethics oversight	N/A

Note that full information on the approval of the study protocol must also be provided in the manuscript.

Field-specific reporting

Please select the one below that is the best fit for your research. If you are not sure, read the appropriate sections before making your selection.

☐ Life sciences ☐ Behavioural & social sciences ☒ Ecological, evolutionary & environmental sciences

For a reference copy of the document with all sections, see nature.com/documents/nr-reporting-summary-flat.pdf

Ecological, evolutionary & environmental sciences study design

All studies must disclose on these points even when the disclosure is negative.

Study description	Over a complete spring season, we collected 37,429 hours of audio from 28 networked sensors in forests across Norway. We used machine learning to automatically identify bird vocalizations, and with expert validation found we were able to classify 57 species (14 full migrants) with over 80% precision.
Research sample	The research sample is all birds whose vocalizations were collected across the 37,429 hours of recorded soundscapes.
Sampling strategy	No sampling strategy was used. All bird detections were used to generate plots and train models.
Data collection	Acoustic data were collected on Bugg recording devices. Audio was segmented into 5-minute files and either transmitted via cellular networks or manually collected and uploaded at the end of the study.
Timing and spatial scale	Between April 1 and June 30, 2022, we collected 37,429 hours of audio data from 28 sites in eight regional clusters across Norway. The solar-powered, networked acoustic monitoring stations were installed from Kristiansand on the southern tip of Norway, to Finnmark in the far north, approximately 1,700 km apart.
Data exclusions	To only include high-quality detections, we exclude all recordings where the BirdNET model score was below 0.8.
Reproducibility	All data and code to generate all plots and statistical tests in the study are available on Zenodo.
Randomization	N/A
Blinding	N/A

Did the study involve field work? ☒ Yes ☐ No

Field work, collection and transport

Field conditions	Acoustic recorders were installed in Norwegian forests during the winter and early spring season. In northern areas like Tromsø, the temperature was quite cold and snow often covered the ground. When installing in more southern areas, like Kristiansand and Stavanger, conditions were more mild.
Location	We installed 28 recorders in Norwegian forests across the latitudinal extent of the country. The exact latitude and longitude of each

Location	recorder were randomized by up to 0.01 degrees to protect the location of the Norwegian Breeding Bird Survey sites with which they were related. These locations with added noise are available in the Zenodo database.
Access & import/export	All installations of acoustic recorders were cleared with landowners. Data was either transmitted to a cloud server via wireless networks or were collected and manually uploaded at the end of the study if cell service was unavailable.
Disturbance	Minimal disturbance was caused by the study. Recorders were installed, left in the field for the several month duration of the study, and were then manually retrieved in the autumn.

Reporting for specific materials, systems and methods

We require information from authors about some types of materials, experimental systems and methods used in many studies. Here, indicate whether each material, system or method listed is relevant to your study. If you are not sure if a list item applies to your research, read the appropriate section before selecting a response.

Materials & experimental systems

n/a	Involved in the study	
☒	☐	Antibodies
☒	☐	Eukaryotic cell lines
☒	☐	Palaeontology and archaeology
☐	☒	Animals and other organisms
☒	☐	Clinical data
☒	☐	Dual use research of concern
☒	☐	Plants

Methods

n/a	Involved in the study	
☒	☐	ChIP-seq
☒	☐	Flow cytometry
☒	☐	MRI-based neuroimaging

Animals and other research organisms

Policy information about studies involving animals; ARRIVE guidelines recommended for reporting animal research, and Sex and Gender in Research

Laboratory animals	N/A
Wild animals	Birds and other animals were acoustically recorded at several dozen recording sites across Norway. No animals were caught or directly interacted with in any way.
Reporting on sex	N/A
Field-collected samples	N/A
Ethics oversight	N/A

Note that full information on the approval of the study protocol must also be provided in the manuscript.

Plants

Seed stocks	N/A
Novel plant genotypes	N/A
Authentication	N/A
